# Editorial: Advances in conservation, characterization, and use of asparagus genetic resources

**DOI:** 10.3389/fpls.2023.1332117

**Published:** 2023-11-10

**Authors:** Roberto Moreno, Patricia Castro, Jose V. Die

**Affiliations:** Department of Genetics, Escuela Técnica Superior de Ingeniería Agronómica y de Montes (ETSIAM), University of Córdoba, Córdoba, Spain

**Keywords:** asparagus, genetic resources, bioactive compounds, GWAS, sex determination, mitochondrial, breeding

Garden asparagus (*Asparagus officinalis* L.) is one of the most important crops worldwide, grown in a variety of climatic conditions. Since modern cultivars share a common origin in the ‘Violet Dutch’ population Geoffriau et al., 1992, their genetic base is narrow. This limitation hampers the development of new hybrids. Historically, total yield has been the fundamental characteristic screened by breeding programs. However, yield can also be determined by quantitative traits, such as the number and diameter of the spears or the quality of the spears, the genetic control of which remains poorly understood García et al., 2023. Furthermore, additional efforts are currently underway to identify resistance to diseases, as many production areas face challenges associated with a range of biotic conditions that limit crop productivity Jacobi et al., 2023; Nothnagel et al., 2021.

To mitigate the negative consequences of the narrow genetic base in the species, several efforts have been focused on preserving and restoring genetic diversity and introducing genetic variation. However, the utilization of genetic resources such as landraces or wild-related species in the development of new varieties lags behind other important vegetable crops Moreno et al., 2021. Several studies have emphasized the valuable potential of these genetic resources as sources of desirable genes for breeding programs due to their tolerance or resistance to a range of biotic and abiotic stresses Kanno and Yokoyama, 2011. Additionally, these genetic resources in this crop may play a role in the development of new varieties with higher levels of bioactive compounds, meeting the current consumer demand.

Through five studies involving 21 researchers, this Research Topic showcases recent advancements across various disciplines pertaining to the preservation, characterization, and utilization of asparagus genetic resources. In the Research Topic, Drost provides a historical overview of asparagus research and opens a discussion on how to refocus international research efforts to breed superior plant materials to meet the challenges of the future. Considering key factors that have characterized asparagus breeding, the environmental influence, and the pivotal role of root systems, the author concludes by strongly advocating the continued pursuit of research to unravel the complexities of spear growth regulation, improve asparagus productivity, adapt the plant for mechanization, and maintain sustainability and profitability.

By supplementing the existing publicly available nuclear and chloroplast sequences, Sheng et al. investigate the mitochondrial genomic sequence and explore its composition through a meticulous structural analysis. The research discusses the plant’s phylogenetic status and evolutionary relationships, providing valuable data for taxonomic studies and the future utilization of garden asparagus germplasm resources. The author’s research initiative bridges a crucial gap in asparagus genomic sequencing, enhances our comprehension of gene exchange between organelle genomes and advances our understanding of mitochondrial genomics.

Concerned by the narrow genetic basis of cultivated asparagus germplasm, Sala et al. investigation centers on the analysis of asparagus germplasm collections, as new sources of variability are pivotal to broaden current gene pool and for incorporating unexploded diversity in future genetic improvement programs. Based on a panel of 378 asparagus genotypes, the authors perform genome-wide association analyses for asparagus quality traits, intensity of anthocyanic coloration in spears and sex determination. This study significantly contributes to the field of genetic diversity research.

Given the superior agronomic performance of male plants in crop conditions, the sex determination system has traditionally been a recurring hot topic. The investigation by Kanno et al. delves deeper into the comprehension of DNA markers for sex identification. Previous research revealed that the available markers were not entirely suitable for identifying sex in the purple cultivar ‘Pacific Purple’. The authors identified two types of male individuals within this cultivar, namely PP-m and PP-m*, based on whether the DNA markers can or cannot identify the sex type. Here, a comparative analysis of one of the sex-determining genes shows that the PP-m and PP-m* types have similar sequences from *A. officinalis* and *A. maritimus*, respectively. These results have implications for deciphering the origin of the development of ‘Pacific Purple’.

Last but not least, the study by Vieira Alcaide et al. focuses on the field of bioactive compounds and presents a valorization of co-products from asparagus cultivation by obtaining extracts enriched in various phytochemicals found in asparagus tissues, particularly flavonoids and saponins. This research promotes sustainable agronomic practices within the framework of a circular economy and the bioactive compounds offer significant techno-functional potential in the agri-food industry.

In summary, this Research Topic highlights recent findings and literature demonstrating the potential and complexity of utilizing garden asparagus germplasm resources. We trust that the studies, the authors’ conclusions, and the content within this Research Topic will be as interesting to you as the editorial process has been for us.

## Author contributions

RM: Writing – original draft, Writing – review & editing. PC: Writing – original draft, Writing – review & editing. JD: Writing – original draft, Writing – review & editing.
